# A Survival-Adjusted Quantal-Response Test for Analysis of Tumor Incidence Rates in Animal Carcinogenicity Studies

**DOI:** 10.1289/ehp.8590

**Published:** 2005-11-10

**Authors:** Shyamal D. Peddada, Grace E. Kissling

**Affiliations:** Biostatistics Branch, National Institute of Environmental Health Sciences, National Institutes of Health, Department of Health and Human Services, Research Triangle Park, North Carolina, USA

**Keywords:** cancer bioassay, Cochran-Armitage trend test, multiple pairwise comparisons, National Toxicology Program, order-restricted inference, poly-3 trend test

## Abstract

In rodent cancer bioassays, groups of animals are exposed to different doses of a chemical of interest and followed for tumor occurrence. The resulting tumor rates are commonly analyzed using a survival-adjusted Cochran-Armitage (CA) trend test. The CA trend test has reasonable power when the tumor-response curve is linear in dose, but it may be underpowered for a nonlinear response. An alternative survival-adjusted test procedure based on isotonic regression methodology has previously been proposed. Although this alternative procedure performs well when the tumor response is nonlinear in dose, it has less power than the CA trend test when the response is linear in dose. Here, we introduce a new survival-adjusted test procedure that makes use of both the CA trend test and the isotonic regression-based trend test. Using a broad range of experimental conditions typical of National Toxicology Program (NTP) bioassays, we conducted extensive computer simulations to compare the false-positive error rate and power of the proposed procedure with the survival-adjusted CA trend test. The new procedure competes well with the survival-adjusted CA trend test when observed tumor rates are linear in dose and performs substantially better when observed tumor rates are nonlinear in dose. Further, the proposed trend test almost always has a smaller false-positive rate than does the survival-adjusted CA trend test. We also developed an order-restricted inference-based procedure for performing multiple pairwise comparisons between each of the dose groups and the control group. The trend test and the multiple pairwise comparisons test are demonstrated using an example from a study conducted by the NTP.

A major responsibility of the National Toxicology Program (NTP) is to investigate the potential toxic and carcinogenic effects of various chemicals. Studies conducted by the NTP are used by the U.S. Environmental Protection Agency, the Food and Drug Administration, and other federal and state agencies in their consideration of regulations and policies for protecting public health. The 2-year rodent cancer bioassay is an important component of the NTP’s investigations; these bioassays typically involve groups of male and female mice and rats randomly assigned to either a control group or one of three dose groups (low, medium, and high). At death, each animal is extensively examined for microscopic and macroscopic tumors, as well as for abnormal changes in tissues. Of particular interest is whether the rate of occurrence of a specific tumor is related to dose.

Because some animals do not survive to the end of the 2-year study, the NTP’s statistical analyses of site-specific tumor rates employ a survival-adjusted, continuity-corrected Cochran-Armitage (CA) trend test, the poly-3 trend test (Armitage 1955; Bailer and Portier 1988; Cochran 1954; Portier and Bailer 1989). For simplicity, in this article we refer to this test as the NTP trend test. Typically, this trend test is followed by pairwise comparisons of each dose group with the control group.

It is important to distinguish between two types of parameters related to the problem of current interest, namely, age-specific tumor incidence rate and lifetime tumor rate. The former is the hazard rate associated with the age at tumor onset, and the latter is the expected proportion of animals that ever develop a tumor (at any age), which is a function of both the tumor incidence rate and the mortality rate. Thus, depending on mortality patterns, lifetime rates generally will not correspond exactly with age-specific incidence rates. The age-specific incidence rate is a more meaningful, but less accessible, end point than is the lifetime rate. For simplicity of notation, we have dropped “age-specific” from the phrase “age-specific tumor incidence rate.”

All point estimators discussed in this article and in other reports, such as Bailer and Portier (1988) and Peddada et al. (2005), estimate lifetime tumor rates. Further, trend tests such as the NTP trend test, the trend test developed by Peddada et al. (2005), and the trend test proposed in this article use survival-adjusted lifetime tumor rates to test for trends in tumor incidence rate. Simulation studies performed by Dinse (1991) and Peddada et al. (2005) and in this article suggest that, although these tests employ lifetime tumor rates rather than tumor incidence rates, they also provide valid inferences about tumor incidence rates.

Although tumor incidence rates may strictly increase with dose, lifetime tumor rates may have an umbrella-shaped or a plateau-shaped dose–response curve because of higher mortality in the upper dose groups. An umbrella or plateau-shaped response curve for lifetime tumor rate is also likely to occur if the tumor incidence rate has a plateau-shaped dose–response curve. In such situations, the NTP trend test may not be sensitive enough to detect this dose-related response because it is based on linear regression of the estimated lifetime tumor rate on dose. For example, in a study of the chemical isoprene, female rats were exposed to 0, 220, 700, or 7,000 ppm isoprene by inhalation for 2 years (NTP 1999). Mammary gland fibroadenomas occurred in 19, 35, 32, and 32 of 50 animals, respectively. Survival did not differ among dose groups, and the survival-adjusted lifetime tumor rates were 44%, 74%, 74%, and 73%, respectively. The NTP trend test yielded a *p*-value of 0.11. However, each of the pairwise comparisons with the control group was significant at *p* < 0.002 (NTP 1999). Data such as these are not uncommon in NTP studies, and in these situations the NTP trend test may fail to detect a significant chemical effect.

Motivated by such examples, Peddada et al. (2005) developed an order-restricted inference-based procedure that is well suited for nonlinear responses. Simulation studies suggest that this isotonic regression-based test performs better than does the NTP trend test when the tumor rates increase nonlinearly in dose (Peddada et al. 2005). However, this test may have less power than the NTP trend test when the tumor response increases linearly with dose. Therefore, in this article we propose a new test that modifies their isotonic regression-based test. Based on our extensive simulations, the resulting test competes well with the NTP trend test for strictly linear dose–response patterns and performs better than the NTP trend test for nonlinear dose–response patterns.

In addition to testing for dose-related trends, NTP performs pairwise comparisons between each of the dose groups and the control group, with particular attention paid to the medium- and high-dose groups. Currently, no adjustment is made for multiple comparisons. Consequently, the NTP’s pairwise comparison tests are subject to false-positive rates that exceed the nominal 0.05 level. Using the order-restricted inference methodology developed in Hwang and Peddada (1994) and Peddada et al. (2001), we introduce a new pairwise comparison procedure that controls the overall false-positive rate.

## Materials and Methods

Suppose there are *K* dose groups with doses 0 = *d*_1_ < *d*_2_ < . . . < *d_K_* with *n_i_* animals assigned to the *i*th dose group, *i* = 1, 2, . . ., *K*. We denote the tumor status at necropsy of the *j*th animal in the *i*th dose group by *y_ij_*, where *y_ij_* = 1 if the animal has a specific tumor and is 0 otherwise. To compute the poly-3 survival-adjusted sample size for the *i*th group (Bailer and Portier 1988; Portier and Bailer 1989), we define animal-specific weights *w_ij_* = 1 if the *j*th animal had a tumor at necropsy, otherwise *w_ij_* = *t_ij_*^3^ where *t_ij_* is the fraction of duration of the study for which the animal survived. For the *i*th dose group, the poly-3 survival-adjusted sample size is


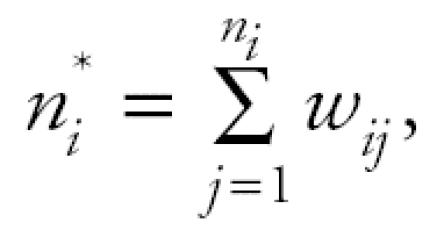


and the poly-3 survival-adjusted estimator for the lifetime tumor rate, π*_i_*, is


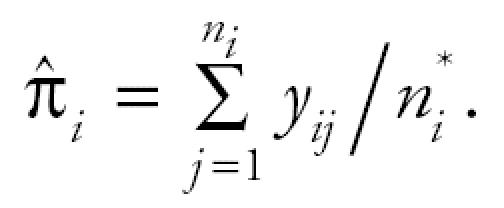


### Test for an increasing trend in dose.

The poly-3 survival-adjusted CA trend test statistic is


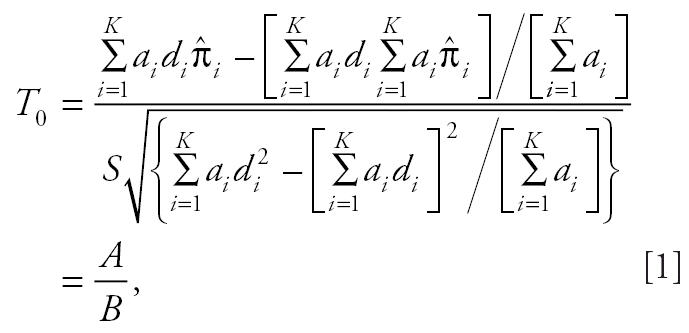


where, for *i* = 1, 2, . . ., *K*,


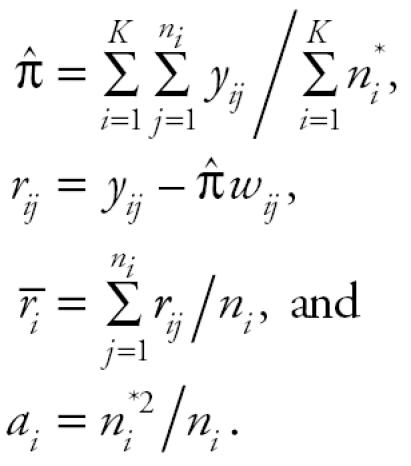


Furthermore,


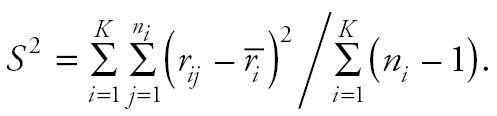


Note that *S*^2^ is a jackknife variance estimator introduced in Bieler and Williams (1993). Performance of the above trend statistic was evaluated by Peddada et al. (2005).

The NTP uses a continuity-corrected poly-3 trend test statistic, defined as


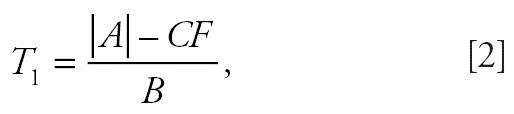


where


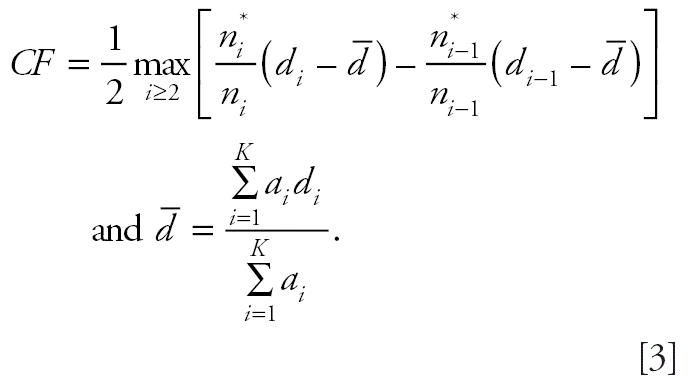


The null hypothesis of no chemical effect on tumor incidence is rejected in favor of the alternative that there is a positive trend in dose if *A* > 0 and *T*_1_ exceeds the (1− α)th percentile of a standard normal distribution.

As an alternative to the poly-3 survival-adjusted CA trend test statistic, Peddada et al. (2005) introduced the following trend test statistic:


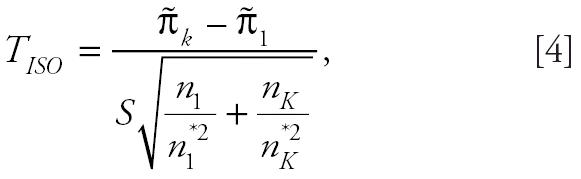


where


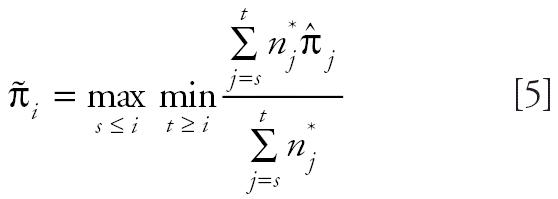


are the isotonized values of 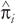 under the order restriction that π*_i_* values are nondecreasing with dose. This statistic performs well in terms of power when π*_i_* values are monotonically, but nonlinearly, increasing with dose, whereas the poly-3 survival-adjusted CA trend test performs well when π*_i_* values are linearly increasing with dose (Peddada et al. 2005).

Motivated by these observations, we propose a hybrid statistic that draws on both of these procedures so that the resulting test statistic has improved power in all situations; that is, the proposed statistic is more likely to detect a dose-related trend, if it exists, than is the poly-3 survival-adjusted CA trend test. First, we note that in some instances it is possible for mortality rates in one or more dose groups to be substantially higher than in the control group. In such cases,


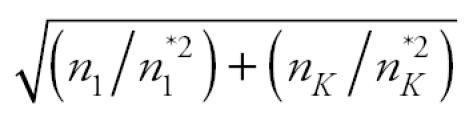


in the denominator of the above test statistic may inflate the false-positive or type I error rates. For this reason, we modify the trend statistic *T_I_*_SO_ of Peddada et al. (2005) as


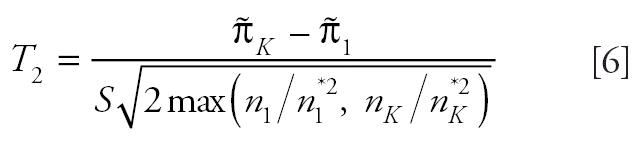


and propose the maximum of the NTP trend test statistic and the modified isotonic regression-based trend statistic,


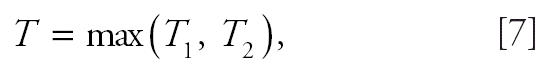


as the test statistic for testing a dose-related nondecreasing trend in tumor incidence rate. Note that *T* is the larger of *T*_1_, a test statistic for the linear trend in survival-adjusted proportions, and larger than *T*_2_, a test statistic for the largest difference in survival-adjusted proportions after standardization to a nondecreasing pattern.

We approximate the distribution of *T* under the null hypothesis that there is no difference in tumor incidence rates among the dose groups as follows. We generate *K* independent standard normal random deviates, *X*_1_, *X*_2_, . . . *X_K_*, and compute


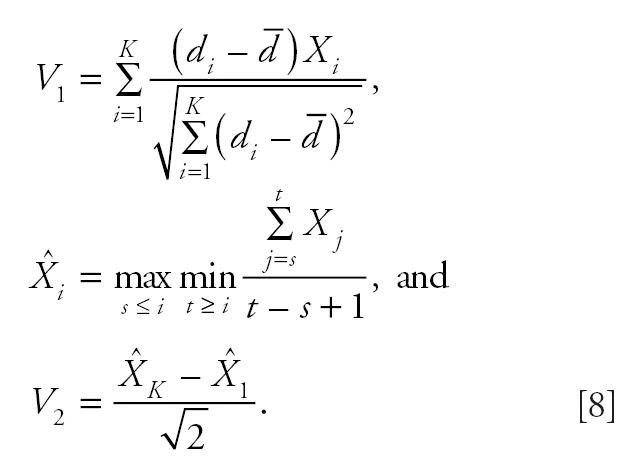


In the above expression,


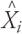
, *i* = 1, 2, . . ., *K*, are the “isotonized” values of *X_i_* We approximate the null distribution of *T* by simulating the distribution of max(*V*_1_, *V*_2_) from which *p*-values or critical values for the trend test can be obtained.

Although all our computations were performed in FORTRAN language, we are in the process of developing a JAVA-based software for implementing the methodology introduced in this article. For additional details regarding the software, please contact the authors.

### Multiple pairwise comparisons with the control group.

In addition to performing a trend test, scientists at the NTP are often interested in performing pairwise comparisons between each of the dose groups and the control group. In this situation, the null hypothesis is that the tumor incidence rates in the dose groups are no larger than the rate in the control group; the alternative hypothesis is that the tumor incidence rate in at least one dose group is strictly larger than in the control group. In order-restricted inference terminology, this alternative hypothesis is known as simple tree order. Currently, the NTP performs pairwise comparisons of each dose group with the control group by applying test statistic *T*_1_ on two groups, the control group and the particular dose group of interest. Although comparisons are generated between the control group and each of the dose groups, NTP researchers are particularly interested in whether the tumor incidence rate in either of the top two dose groups exceeds the rate in the control group. This approach does not account for multiple comparisons between each of the dose groups and the control group. In the following, we propose a simple test statistic derived from the order-restricted inference methodology introduced by Hwang and Peddada (1994) and used by Peddada et al. (2001).

Using the procedure described by Hwang and Peddada (1994), lifetime tumor rates for the dose groups are estimated by 
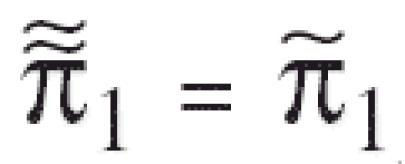
 for the control group, and 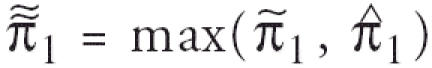, *i* ≥ 2.

To illustrate the above formula, suppose the poly-3 tumor rates for the four test groups are 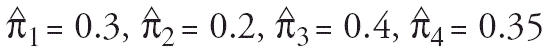, with corresponding poly-3 adjusted sample sizes of *n*_1_^*^ = 44.2, *n*_2_^*^ = 45.6, *n*_3_^*^ = 40, *n*_4_^*^ = 41, respectively. Then, using the formula (Equation 5) for 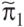 we have


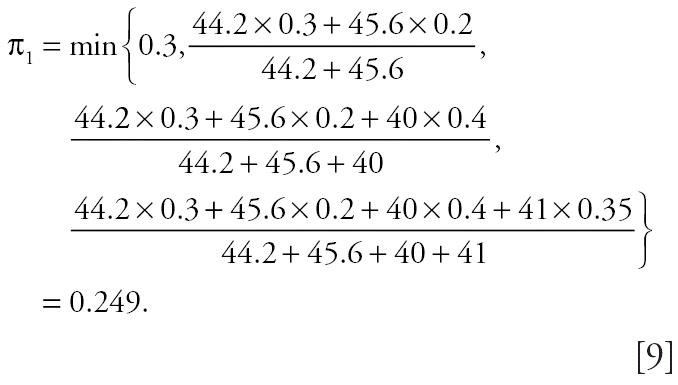


Accordingly, 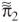 = max(0.249, 0.2) = 0.249, 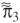 = max(0.249, 0.4) = 0.4, 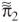 = max(0.249, 0.35) = 0.35.

To derive the test statistic and its distribution under the null hypothesis that all dose groups have the same tumor rates, we first generate *K* independent standard normal random deviates *X*_1_, *X*_2_, …, *X_K_*. Let


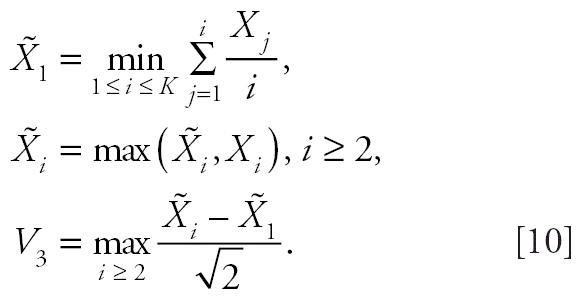


The proposed test statistic for comparing the *i*th dose group with the control group is


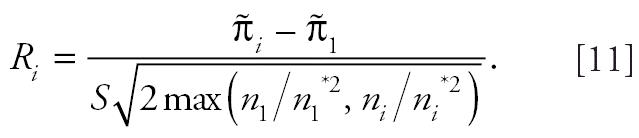


Approximate critical values for *R*_2_, *R*_3_, . . ., *R_K_* can be obtained from the simulated distribution of *V*_3_. The proposed pairwise procedure rejects the null hypothesis if *R* = max(*R*_2_, *R*_3_, . . ., *R_K_*) exceeds the critical value derived from the distribution of *V*_3_. This is a nonparametric analogue of Tukey’s honestly significant differences post hoc multiple comparisons procedure commonly applied in analysis of variance settings.

## Results

### Simulation study.

We conducted an extensive simulation study to compare the performance of the proposed trend test, *T*, with the NTP trend test. A total of 750 nonnull configurations and 150 null configurations, similar to those commonly encountered in the NTP rodent bioassays, were simulated. All simulation results reported in this article are based on 10,000 simulation runs, and the nominal level of significance is α = 0.05.

Simulation parameters were patterned after the NTP rodent cancer bioassays. We considered a total of three dose groups (low, medium, and high) and a control group, with 50 animals assigned to each group. As described by other authors (e.g., Dinse 1991; Peddada et al. 2001, 2005), for each animal in the *i*th dose group, *i* = 1, 2, 3, 4, we generated realizations of two independent Weibull random variables, *Y_i_*_1_ and *Y_i_*_2_, where *Y_i_*_1_ represented the time to tumor onset and *Y_i_*_2_ represented the time to death from natural causes. The survival function of *Y_ij_*, *i* = 1, 2, 3, 4 and *j* = 1, 2 is given by *P*(*Y_ij_* > *t*|d*_i_*) = exp(−ψ*_j_*φ*_ij_^di^t*^γ^*^j^*). We simulated these random variables such that the duration of the study was 24 months, which is typical of the NTP rodent bioassays. We simulated two dose patterns, 2-fold dose spacing and 5-fold dose spacing, namely, (*d*_1_, *d*_2_, *d*_3_, *d*_4_) = (0, 0.5, 1, 2) and (0, 0.1, 0.5, 2.5).

As previously described (Peddada et al. 2005), we considered constant dose effect on mortality; that is, φ*_i_*_2_ = φ_2_, with patterns of φ_2_ = 1 (no effect), 1.5, 2, 2.5, and 3 (severe effect). We set the mortality shape parameter at γ_2_ = 5 and baseline mortality scale parameter at ψ_2_ = 4.479 × 10^−8^ so that 70% of the animals in the control group survived to the end of the 2-year study, a rate often observed.

The three tumor onset shape parameter (γ_1_) values considered in this study were 1.5, 3, and 6. Poly-3 survival adjustments are based on the assumption that the true tumor onset is Weibull with shape parameter γ_1_ = 3 (Portier and Bailer 1989). Thus, the ideal situation for the poly-3 survival correction is γ_1_ = 3. We considered five different background tumor rates, π_1_, ranging from rare (0.001, 0.01, 0.05) to common (0.15, 0.30). Values of the baseline tumor onset scale parameter, ψ_1_, corresponding to each π_1_ are given in Table 1. Finally, we chose six different sets of the effect of dose on tumor onset, φ*_i_*_1_, for each of the five background tumor rates; values of φ*_i_*_1_ are given in Table 2. In each case, the null hypothesis corresponds to the case when the incidence rates are all equal; that is, the ratios are (1:1:1:1). Thus, a total of 375 nonnull and 75 null configurations were considered for each of the two dose spacings.

**Figure 1:**
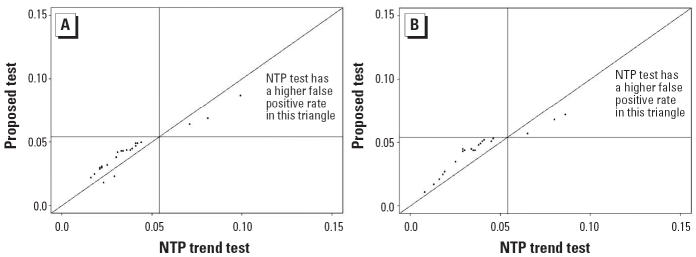
Comparison of false-positive error rates for 2-fold dose spacing (*A*) and 5-fold dose spacing (*B*). The false-positive error rate of the proposed trend test was plotted against the false-positive error rate of the NTP trend test. We plotted only the cases where the two procedures differed significantly in terms of false-positive error rates; results are based on 10,000 simulation runs per configuration. The plot suggests that the false-positive rate of the proposed test is always less than that of the NTP trend test when both tests exceed the nominal level.

**Figure 2:**
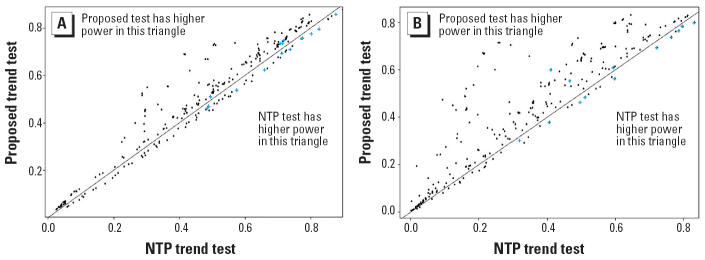
Comparison of power for 2-fold dose spacing (*A*) and 5-fold dose spacing (*B*). In each panel the power of the proposed trend test was plotted against the power of the NTP trend test. Plus symbols (+) indicate cases in which the false-positive error rate of the NTP trend test exceeds both the false-positive error rate of the proposed trend test and 0.05. We plotted only the cases where the two procedures differed significantly in terms of power; results are based on 10,000 simulation runs per configuration. In both (*A*) and (*B*), the “bow”-shaped pattern pointing in the northwest direction, with very few points below the diagonal line, suggests that the proposed test has generally higher sensitivity to detect real trends than the NTP trend test. The gains are substantial as the dose spacing increases from 2- to 5-fold.

**Figure 3:**
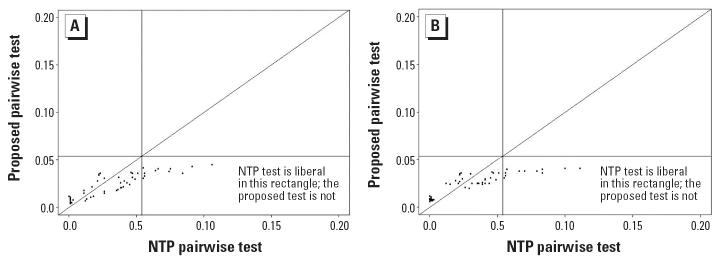
Comparison of false-positive error rates for pairwise tests for 2-fold dose spacing (*A*) and 5-fold dose spacing (*B*). The false-positive error rate of the proposed pairwise comparisons procedure was plotted against the false-positive error rate of the NTP pairwise comparisons procedure for comparing the two highest dose groups against the control. We plotted only the cases where the two procedures differed significantly in terms of false-positive error rates; results are based on 10,000 simulation runs per configuration. In both (*A*) and (*B*), all points are below the horizontal line at 0.054. This suggests that the false-positive rate of the proposed procedure is almost always less than that of NTP pairwise procedure and never exceeds the nominal 0.05 significance level.

Results of the simulation study are represented by scatter plots of false-positive error rates (or power) with the NTP procedure on the horizontal axis and the proposed procedure on the vertical axis. For the trend tests, false-positive error rates are summarized in Figure 1 and powers in Figure 2. For the pairwise comparison procedure, false-positive error rates are summarized in Figure 3. In each case, the diagonal line represents the line of equality between the two tests. The horizontal and vertical lines in Figures 1 and 3 are drawn at a distance of 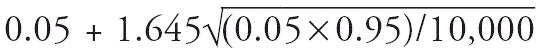 from the origin. In Figures 1 and 3, points falling to the right of the vertical line indicate instances in which the NTP procedure exceeds the nominal level of 0.05, and points falling above the horizontal line correspond to instances in which the proposed test exceeds the nominal level of 0.05. In Figure 2, points falling below and to the right of the diagonal line correspond to instances in which the NTP trend test has more power than the proposed trend test, whereas points falling above and to the left of the diagonal line correspond to instances in which the proposed trend test has more power than the NTP trend test. To reduce clutter in the plots, we tested equality of the false-positive error rates (or power) of the NTP procedure and the proposed procedure using a two-sample *z*-test for proportions, and we plotted only those points for which there was a significant difference between the NTP test and the proposed test at the 5% level of significance.

For the 75 null patterns considered in this simulation study, there were 23 patterns where the two tests had significantly different false-positive error rates (Figure 1). This result was observed for 2-fold spacing as well as 5-fold spacing. The proposed test was rarely more liberal than the NTP trend test when both tests exceeded the nominal level; that is, the false-positive rate of the proposed test never exceeded that of the NTP trend test. Furthermore, the NTP trend test was more liberal than the proposed test for common tumors (π_1_ ≥ 0.15) considered in this study. Although we only plotted the cases for which the false-positive error rates of the two tests differed significantly, the false-positive error rate of the proposed trend test never exceeded 0.087, and that of the NTP trend test never exceeded 0.099.

The power of the two tests differed significantly in 270 of the 375 nonnull dose patterns for 2-fold spacing (Figure 2A). In approximately 70% of these 270 patterns, the proposed trend test had higher power than did the NTP trend test. Thus, a large number of points in Figure 2A are above the diagonal line. Further, in 15 of the 270 patterns (about 6%), the false-positive error rate of the NTP trend test exceeded the nominal 0.05 significance level and was significantly higher than that of the proposed test. These cases are denoted by a “+” in Figure 2A. The gain in power for the proposed test was as high as 0.275 (0.69 for the proposed test vs. 0.415 for the the NTP trend test), a relative gain of 66%. In contrast, the best gain observed for the the NTP trend test was 0.048 (0.502 for the the NTP trend test vs. 0.454 for the proposed test), a modest relative gain of < 10%.

The power gains made by the proposed test were even more substantial for 5-fold dose spacing (Figure 2B). Power of the two tests differed significantly in 264 of the 375 nonnull patterns, and the proposed test had higher power in almost 85% of these patterns. Thus, most points in Figure 2B are above the diagonal. Further, as we observed with the 2-fold dose spacing, in 13 of the 264 patterns (about 5%) the false-positive error rate of the the NTP trend test exceeded the nominal 0.05 significance level and was significantly higher than that of the proposed test. As in Figure 2A, these cases are denoted by a “+.” The gain in power for the proposed test was as high as 0.460 (0.671 for the proposed test vs. 0.211 for the NTP trend test), > 300%. In contrast, the best gain observed for the NTP trend test was 0.038 (0.331 for the NTP trend test vs. 0.293 for the proposed test), a modest relative gain of < 12%.

In cases for which tumor incidence rates increased monotonically, but not linearly, with dose, the proposed trend test performed better than the NTP trend test in terms of both power and false-positive error rate. As expected, the NTP trend test performed better than the proposed test in cases for which tumor incidence rates increased linearly with dose. But even in such cases, the gains made by the NTP trend test were modest. Furthermore, the false-positive error rate of the NTP trend test often exceeded the nominal 0.05 significance level.

For the null configurations described above, we also compared false-positive error rates of the proposed pairwise comparisons procedure with the NTP procedure for pairwise comparisons between the medium- and high-dose groups with the control group. Figure 3 shows that the proposed method maintained false-positive error rates at or below the nominal 0.05 level, whereas the NTP procedure was often liberal, exceeding the nominal level of 0.05. Although we plotted only the cases in which the false-positive error rates of the two tests differed significantly, the proposed pairwise test never exceeded 0.05, whereas the NTP pairwise test had false-positive error rates as high as 0.11.

### An NTP example.

As part of an NTP bioassay on isoprene, female F344/N rats were exposed to isoprene for 2 years through inhalation (NTP 1999). Isoprene is a naturally occurring compound in plants, as well as a byproduct of ethylene production. It is similar in structure to 1,3-butadiene, a potent rodent carcinogen.

Fifty female rats were exposed to 0, 220, 700, or 7,000 ppm isoprene; 19, 35, 32, and 32, respectively, developed mammary gland fibroadenomas. Survival-adjusted tumor proportions showed a plateau-shaped response, with 44%, 74%, 74%, and 73%, respectively, of the animals developing fibroadenomas. The NTP trend test gave a *p*-value of 0.105, whereas each dosed group differed from the control group at *p* < 0.002. Because of the wide dose spacing and the plateau-shaped response beginning at the low dose of 220 ppm, the NTP trend test was not sensitive enough to detect the dose-related response.

The proposed trend test provided a significant dose-related trend in mammary gland fibroadenomas with a *p*-value of 0.0014. As indicated in our simulation study discussed above, this statistic is capable of detecting monotonic nonlinear trends with dose and is not affected by wide dose spacing. Furthermore, using the proposed method for pairwise comparisons, each dose group differs from the control group at *p* < 0.005. From our simulations, we can be confident that, among all of the pairwise comparisons with the control group, the overall false-positive rate of 0.05 is not exceeded.

## Discussion

We have presented a trend test for tumor incidence data that takes advantage of the strengths of the CA trend test when the dose–response relationship is linear and also takes advantage of strengths of nonparametric order-restricted methods when the dose–response relationship is monotonic but nonlinear. Most important, the false-positive rate of the proposed test rarely exceeds that of the NTP trend test when both tests exceed the nominal level, yet in many instances the proposed test outperforms the NTP trend test in terms of power. Further, we have also provided a simple procedure for performing pairwise comparisons between each of the dose groups and the control group;, this procedure controls the overall false-positive error rates when conducting multiple tests.

Because NTP rodent bioassay data are used by federal and state agencies to assist in formulating regulatory policies, it is crucial for the statistical methods to be powerful enough to detect dose-related trends when they exist. Equally important, these methods should not produce excessive false-positive findings. The trend test and the multiple comparisons procedure that we have proposed here make important steps in both directions. When dose–response relationships are monotonic but nonlinear, the proposed trend test is more powerful than is the NTP trend test. Although both trend tests can exceed the nominal 0.05 level under some circumstances, the false-positive error rate of the proposed trend test is almost always less than that of the NTP trend test when both tests exceed the nominal level. Therefore, the occurrences of false positives will be reduced with use of the proposed trend test. Furthermore, the NTP pairwise comparisons method does not correct for multiple comparisons and often exceeds the nominal 0.05 level in pairwise comparisons of each of the dose groups to the control group, particularly for common tumors. The proposed pairwise comparisons method controls the false-positive rate so that it stays near (or less than) 0.05 under a wide range of situations that we commonly encounter in NTP studies. Thus, these proposed methods should provide more accurate decisions about the potential carcinogenic effects of chemicals.

## Figures and Tables

**Table 1:** Patterns of tumor onset shape parameter (γ_1_), and tumor onset scale parameter (ψ_1_) by background tumor rate (π_1_).

	Background tumor rates (π_1_)				
Variables	0.001	0.01	0.05	0.15	0.30
γ_1_ = 1.5
ψ_1_	9 × 10^−6^	9 × 10^−5^	4.7 × 10^−4^	15 × 10^−4^	33 × 10^−4^
γ_1_ = 3
ψ_1_	8 × 10^−8^	8 × 10^−7^	4.2 × 10^−6^	13.4 × 10^−6^	29.7 × 10^−6^
γ_1_ = 6
ψ_1_	6.5 × 10^−12^	6.5 × 10^−11^	32.5 × 10^−11^	10.4 × 10^−10^	23.2 × 10^−10^

**Table 2:** Patterns of tumor incidence ratios (φ_11_:φ_21_:φ_31_:φ_41_) for the four dose groups by background tumor rate (π_1_).

	Tumor incidence ratio (φ_11_:φ_21_:φ_31_:φ_41_)		
Dose–effect set	Very rare tumors (π_1_ = 0.001, 0.01)	Somewhat rare tumors (π_1_ = 0.05)	Common tumors (π_1_ = 0.15, 0.30)
1	1:1:1:1	1:1:1:1	1:1:1:1
2	1:1:1:10	1:1:1:4	1:1:1:2
3	1:1:10:10	1:1:4:4	1:1:2:2
4	1:10:10:10	1:4:4:4	1:2:2:2
5	1:5:5:10	1:1.5:1.5:4	1:1.5:1.5:2
6	1:5:10:15	1:2:3:4	1:1.25:1.75:2
